# Capturing Children's Voices in Healthcare: Development of the PREM‐Ped Questionnaire

**DOI:** 10.1111/apa.70674

**Published:** 2026-07-09

**Authors:** E. Broström, J. Granhagen Jungner, C. Bartholdson, S. Norgren, M. Wenemark

**Affiliations:** ^1^ Department of Women's and Children's Health Karolinska Institutet Stockholm Sweden; ^2^ Astrid Lindgren Children's Hospital, Karolinska University Hospital Stockholm Sweden; ^3^ Department of Health, Medicine and Caring Sciences, Faculty of Medicine and Health Sciences Linköping University Linköping Sweden; ^4^ Unit for Public Health and Statistics East Region Linköping Sweden

**Keywords:** paediatric healthcare, patient‐reported experiences measures (PREMs), psychometric evaluation, questionnaire development

## Abstract

**Aim:**

Patient‐reported experience measures (PREMs) capture how care is experienced and are essential quality indicators in paediatric healthcare, supporting children's right to be heard. Compared with patient‐reported outcome measures (PROMs), which assess health status and well‐being, PREMs are less developed, particularly for children. This study aimed to describe the development and initial psychometric evaluation of PREM‐Ped, a short, child‐friendly questionnaire designed to capture children's own healthcare experiences.

**Methods:**

PREM‐Ped was developed using a multi‐step, participatory process. Content validity was established through a focus group with children who had experience of paediatric healthcare. The questionnaire was refined through four iterative rounds of cognitive interviews. A digitally administered pilot study was conducted in out‐ or inpatient units at Astrid Lindgren Children's Hospital in Sweden during spring 2025. Children aged 4–15 years completed the PREM‐Ped after care (*n* = 586). Qualitative data were analysed thematically. Validity was examined by comparing experiences in acute and elective care. Psychometric properties were evaluated using a unidimensional Rasch measurement model.

**Results:**

The development process resulted in a short, comprehensible questionnaire suitable for children and adolescents. Initial psychometric evaluation demonstrated satisfactory measurement properties. Validity was supported, with PREM‐Ped distinguishing between acute and elective care settings.

**Conclusion:**

We developed PREM‐Ped, a short, child‐friendly questionnaire that enables meaningful measurement of children's healthcare experiences and may support benchmarking and systematic child‐centred quality improvement.

## Summary

- Paediatric healthcare lacks short, child‐friendly patient‐reported experience measures that capture children's own perspectives rather than relying on caregiver proxies.
- A participatory, multi‐step process resulted in a valid and reliable patient‐reported experience measure for children, demonstrating psychometric properties and the ability to distinguish between acute and elective care.
- This instrument enables systematic, child‐centred evaluation and benchmarking of healthcare experiences, supporting quality improvement and further validation across settings and countries.

**Abbreviations**
ECHO: European children's hospital organization
HCPs: healthcare professionals
PREM‐Ped: patient‐reported experience measures in paediatrics
PREMs: patient‐reported experience measures

## 1 Introduction

Patient‐reported experience measures (PREMs) serve as critical quality indicators for healthcare professionals (HCPs), policy‐ and decision‐makers and are vital tools for evaluating paediatric healthcare from the patient perspective [1, 2, 3, 4, 5]. While patient‐reported outcome measures (PROMs) capture patients' health status, functioning, and well‐being, PREMs focus on how healthcare is experienced and delivered. Importantly, PROMs are more extensively developed and standardized, whereas PREMs remain less established [6].

Article 12 in The United Nations Convention on the Rights of the Child [7] asserts that children have the right to express their views in all matters concerning them. Major efforts have been made to develop paediatric PREMs targeting children and adolescents [8, 9, 10]. This is an important endeavour in order not only to generate valid outcomes regarding the quality indicators in healthcare but also to live up to the children's right to be heard in matters concerning them.

However, applying PREM in paediatric settings encounters specific challenges [2, 11]. Firstly, PREMs in paediatric healthcare frequently rely on caregivers to serve as proxies for children, which may not be equivalent to the child's own experiences [2, 12]. Moreover, the instruments are often not tailored to children's cognitive and emotional capacities, resulting in the use of questions originally designed for adults, and PREM surveys traditionally have also been excessively long, limiting their feasibility in clinical settings [13]. Lastly, the use of different PREMs across countries, hospitals, and clinics has challenged the comparability of data, thereby reducing their utility for benchmarking and quality improvement [5, 11].

For paediatric PREMs to be truly effective they need to be developed together with children and adolescents with experiences of paediatric healthcare [8, 9, 10, 14]. In addition to being user‐friendly for children and adolescents, it is crucial that it also shows sound psychometric properties. Rasch analysis provides a sample‐independent measurement framework to examine item fit to a unidimensional construct, evaluate response category functioning, and transform ordinal scores into interval‐level measures [15]. The aim of this study was to describe the development and initial psychometric evaluation of the short, child‐friendly PREM‐Ped questionnaire. The questionnaire aims to be applicable to both outpatient and inpatient care settings, at both national and international levels, ensuring comprehensive and meaningful insights into the healthcare experiences of children and adolescents.

## 2 Methods

### 2.1 Questionnaire Development Process

The development of the PREM‐Ped followed a multi‐step process. An expert group was established consisting of researchers in paediatrics and survey methodology as well as representatives from the national patient survey at Stockholms' Municipalities and Regions (SKR). To gain broad understanding about the field, the group reviewed and synthesized existing PREM questionnaire findings in a study using rapid evidence assessment [16].

The initial version of the questionnaire was discussed in a focus group with children with experience of paediatric healthcare to establish content validity. To further refine the questionnaire, four rounds of cognitive interviews were conducted, with iterative revisions made between the rounds. A pilot study was then carried out at two hospitals in the Stockholm Region.

General principles were that the questionnaire needed to be short, relevant and easy to understand and respond to by children. In developing the questions, particular attention was paid to ensuring that the questions were easy for children to understand and respond to. The response options were chosen to match what children would naturally respond to the questions. Two open‐ended questions were added to give the children opportunities to add things important to them in a non‐directive and inviting manner, encouraging response without exerting pressure.

### 2.2 Participants and Data Collection

**TABLE 1:** Demographic data of the focus group, cognitive interviews and pilot test.

	Focus group	Cognitive interviews	Pilot study
Number of participants	4	23	586
Age mean (range)	15.0 (13–17)	11.5 (4–17)	9.1 (4–15)
Gender (female) (%)	75.0	56.5	45.7
Sick or injured more than 3 months (%)	100	65.2	18.6
Born in Sweden (%)	100	82.0	87.7

#### 2.2.1 Focus Group

The focus group interview was conducted with four participants, aged 13–17, all of whom had substantial experience as patients at Astrid Lindgren Children's Hospital in Sweden. During the focus group interview, the interviewer (CB) presented each of the initial five key topics, each aligned with one of the three domains: information, participation, and care approach, and invited the participants to reflect freely on the questions. The interview concluded with an open discussion about any aspects the participants considered lacking from their perspective and their thoughts on what is important in a short and child‐friendly PREM. The focus group interview was recorded and transcribed verbatim (Table 1).

#### 2.2.2 Cognitive Interviews

Four rounds of cognitive interviews were conducted with 23 children, aged 4–17. The participating children had a wide range of healthcare problems and varied experience of being a patient in paediatric healthcare. Children and their parents were asked about participation by HCPs at Astrid Lindgren Children's Hospital. The majority of the interviews (*n* = 14) were done with one interviewer (CB, JGJ or MW) and one note taker (CB, JGJ, MW or EB) present, while the rest were done with only an interviewer (CB, JGJ or MW). The interviews took place at the patients' room or in a quiet area in the clinic. The interviewer and note taker were not involved in the child's healthcare and were dressed in civilian clothing to create a supportive and safe environment for the child during the interview. The mean interview time was 11 min (range 4–23) not including the time for introduction and signing of consents.

The interview was adapted to each child with the overall aim of helping them to feel relaxed and comfortable during the interview. In the introduction, it was explained to the children that they were helping the research team to test the questions, that there were no “right or wrong” answers to the questions, and if a question was difficult to understand it was a problem with the question and not the child. The interview started with a think‐aloud [17] introduced by an example from the interviewer, followed by probing. The children could decide if they wanted to read the questions themselves or if they wanted a parent or the interviewer to read out the questions. Probes used were for example “Can you tell us more about that?” For the younger children probes were asked after each question to make it easier for them to relate the probe to the question they just answered. The interview ended by asking whether the child thought any important questions were lacking and parents were invited to share their thoughts on the questions. The interviews were recorded and transcribed verbatim.

#### 2.2.3 Pilot Study

Children and adolescents being cared for at out‐ or inpatient units at Astrid Lindgren Children's Hospital between 2025‐01‐15 and 2025‐05‐31 received the final version of the PREM‐Ped questionnaire 24 h after the visit through a digital channel for healthcare communication; the Swedish healthcare guide 1177. The format for questionnaires in this channel required an answer to each question.

The questionnaire was distributed through the guardians 1177 inbox for children aged 4–12 years with a request to let the child answer the questions by themselves, or for the younger children to answer the questions together with the child. Adolescents aged 13–15 years received the questionnaire in their own 1177 inbox. An algorithm prevented a child from get more than one questionnaire during a 3‐month period. The questionnaire included background questions on age, gender, diagnosis > 3 month, born in Sweden or abroad and for inpatient care two additional questions about food and activities.

### 2.3 Data Analysis

Data from the focus group interview were analysed using a deductive thematic approach, guided by Braun and Clarke [18, 19, 20] to identify, analyse, and report patterns in the data. The analysis involved familiarization with the transcribed material, generation of codes linked to the five key topics in the initial questionnaire, and development of subthemes and themes.

The results from the cognitive interviews were analysed to identify problems in the four‐step cognitive process of understanding, retrieval of information, estimation and responding [21].

Pilot study data were analysed using descriptive statistics in IBM SPSS Statistics Version 31.0.2.0. Known‐group validity was evaluated by comparing acute and elective care with the hypothesis that acute care would have lower perceived satisfaction. Differences in proportions were tested using chi‐square test. Psychometric properties were evaluated by using a unidimensional Rasch measurement model for ordered categories in RUMM2030 version 5.8.1 following established recommendations regarding model evaluation [15, 22, 23, 24]. Analyses were based on five class intervals with approximately 30 participants per interval (range 27–70). Overall model fit was assessed using item‐trait interaction statistics (χ^2^‐test) and by item‐person interaction fit residuals (expected mean≈0, SD≈1). Item‐fit is evaluated through visual inspection of Item Characteristic Curves (ICC), standardized fit residuals (±2.5) and *χ* ^2^‐test comparing observed and expected responses. Response category functioning was examined for disordered thresholds. Person fit was assessed by the proportion of residuals exceeding ±2.5. Unidimensionality [25] was evaluated using principal component analysis (PCA) of residuals with a cut‐off of 2.0 for the first eigenvalue (Varimax rotation). Local dependency was assessed using item residual correlations (> 0.15 above the average). Targeting was evaluated by examining the correspondence between item difficulty and the distribution of person ability along the latent trait continuum. Reliability was evaluated using Person Separation Index (PSI) with values > 0.7 indicating acceptable reliability. Differential Item Functioning (DIF) was tested for age (4–10 vs. 11–15 years), gender, type of care (acute/elective), hospital (A/B), visit type (inpatient/outpatient), country of birth (Sweden/other) and illness duration (< 3 vs. ≥ 3 months).

## 3 Results

Aspects identified in previous research (16) included for example: satisfaction with care, information, communication, involvement, healthcare professionals' approach and support for emotions and feelings. These were synthesized into three domains—information, participation, and care approach—which formed the basis for five key topic areas discussed in the focus group (Figure 1).

**FIGURE 1:**
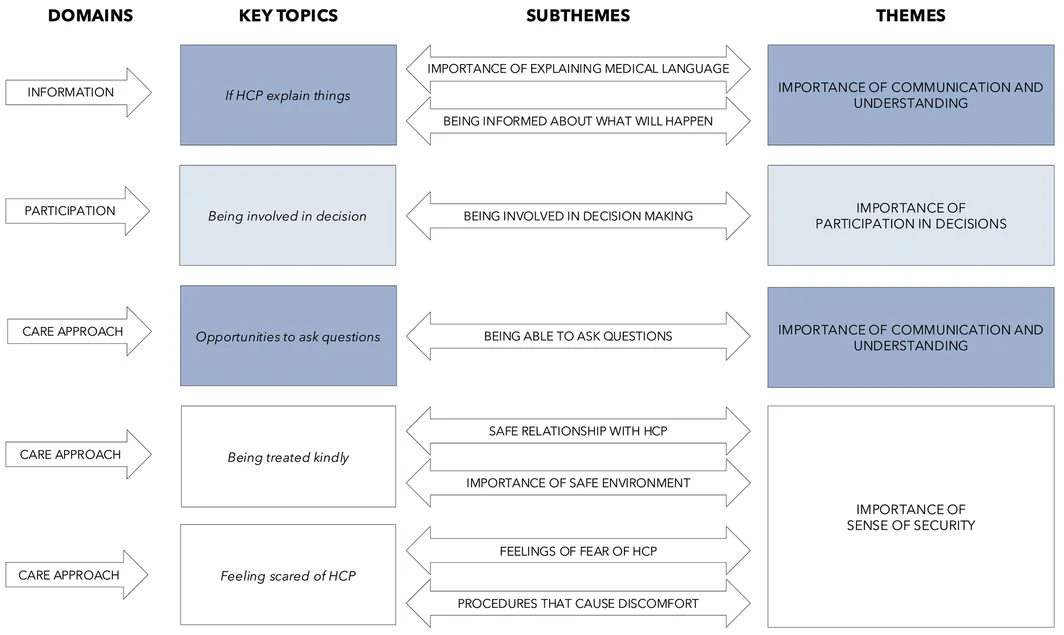
Overview of domains, key topics, subthemes and themes.

### 3.1 Focus Group

The thematic analysis generated three themes (Figure 1). The theme *Importance of communication and understanding* reflects participants' strong desire for clear, age‐appropriate information from HCPs. They emphasized the need for medical language to be explained in a way that they can understand, and they expressed a wish for HCPs to speak directly to them rather than communicating solely with their parents. Participants also highlighted that being informed about the next steps in their care was essential for them to feel prepared and less anxious. Some participants described hesitancy to ask questions when they did not feel comfortable with the HCPs and therefore appreciated when the HCPs actively encouraged questions.

The theme *Importance of participation in decisions* illustrates the participants' wish to be involved in both major and minor decisions regarding their care. They expressed a need to have their opinions respected and to feel that they had the option to decline when something did not feel right for them.

The theme *Importance of sense of security* captures several factors influencing how safe children and adolescents felt in healthcare settings. A trusting relationship with HCPs, characterized by genuine interest and empathy, as well as engagement in conversations about their daily lives, contributed to a sense of safety. Participants described that they sometimes felt afraid of certain HCPs, which negatively affected their experience. The participants also distinguished between fear and the discomfort caused by medical procedures. They felt that the concept of “fear for a specific HCP” did not adequately capture the physical and emotional discomfort associated with such procedures.

### 3.2 Cognitive Interviews

In the first round a five‐point scale (Always/Often/Sometimes/Rarely/Never) was used. An additional “Don't know” option was used in all versions and rounds. When the response options were read aloud to younger children, some had difficulty remembering them and they often had to be repeated. A few children in that situation chose “Don't know.” Therefore, in subsequent rounds, the response scale was reduced to four options. As only one child selected “Rarely” in round one, the revised scale was (Always/Often/Sometimes/Never).

Some children spontaneously expressed their answers in terms of good or bad (e.g., “I say Always because I think they explain well” [G17]; “Sometimes they are OK but sometimes they are bad at explaining things to me.” [G7]). In round two, the response options for questions 2 and 3 were therefore changed to (Good/Quite good/Quite bad/Bad). As this appeared easier and more natural for the children, a similar format was subsequently applied to all questions. In the final refinement, “Very” was added to the first and last option, reflecting wording spontaneously used by the children (e.g., “They have explained what diabetes is and it has been really good.” [B15]). The question on friendliness was perceived as the easiest to understand also for younger children and was therefore placed first from round 2 onward. See further in Table 2.

**TABLE 2:** Results and considerations from cognitive interviews.^a^

Final question	Interpretation	Considerations
Were the healthcare staff friendly to you? Response options: Very friendly Quite friendly Not so friendly Not friendly at all Don't know	Aim: To measure whether the child feels that the HCP treat them well and respectfully The children described that the HCP were positive, engaged in small talk and showed that they care, treated them well and spoke respectfully They said I'm smart and nice and gave me some things. G6 They are very kind. They ask me what I will do in the weekend, about school and stuff. B11:1 Yeah, but I mean, no one has been rude. Everyone is, like—what's it called—they talk with respect or something. No one has been unkind. G17:2 They're always positive and all that. And they treat me well when I'm here. There are no sour faces‐ just happy. G17:1 I think they are pretty kind, but sometimes they're a little not so nice. How are they then? Then they're a bit stressful and not so nice. G7	Some adults were concerned that children who must go through difficult medical procedures would see the HCP as unkind or mean. However, no child expressed this. The children still felt that the HCP were kind and helped them, especially if the HCP took the child's needs into account during the procedure This was followed up with a probe in some interviews. Do you feel they are kind even if they do things that feels difficult for you or hurts? Yes, they're just helping me. B11:2 Yes, because they, like… they held my hand and showed that they were really there. And they respected if you said no. G17:2
How good were the healthcare staff at explaining things to you? Response options: Very good Quite good Quite bad Very bad Don't know	Aim: To measure the HCP's ability to explain things to the childThe children described that the HCP explained what they were doing and about their medicines. They also gave examples of showing a video about how an examination will be carried out, so that they could be prepared. When the child didn't understand, they wanted the HCP to explain more or in a different way. Lower ratings were given when the child still didn't understand or when there were many difficult words that weren't explained. One child mentioned that the HCP should address the child directly and not just the parents. Both quantity and quality of explanations were mentioned It was very good. Do you remember anything they explained to you? That they should shine a light into the eyes and things like that. B10 I think they are quite good but not very. I don't understand much. How do you want them to explain? I want them to show… that's when I understand best—when they show me. G6 Sometimes it's ok but sometimes they are bad at explaining things to me. And what is it like when someone is bad at explaining? Then I don't think they explain very much or very good. G7	The aim was to focus on the HCP's ability to explain, to avoid that the children would evaluate their own ability to understand. Some PREM questionnaires ask whether the patient understood the information, which we wanted to avoid not to risk that the children feel incompetent. Most children seemed to relate to the HCP's ability to explain A few children still related to if they understand or not, but no one seem to blame their own ability for not understanding I think they were very good. I understood. G13 Sometimes I didn't understand. B11:1
How good were the healthcare staff at listening to you? Response options: Very good Quite good Quite bad Very bad Don't know	Aim: To measure patient‐participation The question captured multiple dimensions of feeling listened to. Some children provided examples where HCP asked about their well‐being or pain, that they waited until the child felt ready for a procedure, they felt anxious about or responded when the child expressed refusal. Overall, it appears that children perceived themselves as being listened to when HCP actively involved them by for example, asking questions When I should push it into my tummy by myself, I got kinda' scared. But then they, like, waited until I was okay with doing it, you know? So, like, there was no rush. B15 They are really good because they want to listen to me. Not talk to anyone else or so. G6 She asked how much pain I had like from zero to ten. If the pain was more than three, I should press the button and they will come. They often ask what I think. B13	The first version “Did the staff ask you what you think when they were to decide something about your care?” was difficult for the children to understand What do they mean by that? How should I answer if I don't understand? G17:1 A few If the older children understood the question They always ask me what I think if they want to go ahead with a treatment or something. That feels good because then I feel involved. After all, it is about me. G17:1 The revised formulation, focusing on being listened to, appeared to still capture aspects of participation and patient‐centred communication Even adults may find it difficult to answer questions related to participation and decision‐making, as they often rely on the professionals' expertise and may be reluctant to take responsibility for decisions when they perceive they have limited knowledge themselves. For children, this is likely even more challenging, especially if they cannot opt out from procedures they don't like or want to do Well, I think… they probably know their job best. And they probably know what's best for the person. But of course, you should still get to have your say and ask questions and stuff. G17:3
How easy was it to ask the healthcare staff if you were wondering about something? Response options: Very good Quite easy Quite difficult Very difficult Don't know	Aim: To measure if the children felt they were invited and comfortable to ask questions It appeared that children who felt comfortable with the HCPs also felt that they were able to ask questions. It also became easier to ask questions when HCPs explicitly invited the child to do so Very easy. You can just press the button and then you can ask about anything you want. B15 Yes, I could ask. It depends' a little who it is… but most of them I can ask. B13 I think that is very easy. Why is it easy? They ask… I can ask what I want. G13 No, I usually ask my mom because sometimes they, like they don't really understand what I say. G14 Those who do not have questions in some cases respond that they do not know how easy it would be, while others answer the question hypothetically I don't know. I didn't ask them so much. G6 Yes, absolutely. I'm sure I could, but I like had no question. So, I kind of wouldn't know, but I feel I could always ask if I wanted to. B11:1	The phrasing in the first version phrased, “Could you ask,” was problematic, as it placed emphasis on the child's ability to ask questions I don't speak very much. Is that because you couldn't ask because they were stressed or because you felt a bit shy? Yes, I feel a little shy. G10 The question was rephrased to focus on the child's perception that asking questions would be possible, regardless of whether they had actually asked any question. However, several children spontaneously responded “Yes” when the question was presented in a Yes/No format. The question was therefore further revised to align with an Easy/Difficult scale It is possible that some children would never dare to ask questions; however, if HCP communicate effectively, it should nonetheless be a goal to ensure that even shy children feel that they would be able to ask questions
How good were the healthcare staff at helping you if you were worried or scared? Response options: Very good Quite good Quite bad Very bad Don't know	Aim: To measure if the children felt that the HCP could help them if they felt worried or scared The children described things that you may be afraid of at the hospital, as in needle related procedures or sleeping at the hospital. They also gave relevant examples of how HCPssupported them, for example, by waiting until the child felt ready, preparing and explaining procedures, and demonstrating that they were there to help. They are quite good. Like when I was going to press this into the tummy, I didn't dare to do it. But they helped me so I could hold it, but they pressed. B15 They are very good. How did they help you? They said that my head says like this will hurt a lot but it will not. That is what they said and that made me feel better. G6	In the first two versions we tried more simple questions if the child felt scared of the HCP or felt scared during the visit. They were easy to understand but gave answers about being scared of; for example, surgery Yes, a little. The ones who give me medicine, they are not scary. But the doctors they just come in and say just like… harsh. B13 I felt scared because I wondered how it would go [the surgery]. B11:2 The final version therefore focused on the HCP's ability to help the child if being scared or worried. It is technically the most difficult question since it requires the child to identify situations when they were scared and then think of how the HCPs acted

^a^ Quotes from the interviews is in italics.

### 3.3 Pilot Study

The frequency distributions showed that all response options were used although the results were skewed toward the positive direction (Table 3). In total, 41% of the children gave the best answer on all five questions, giving a clear ceiling effect. There were significant differences between “very good” for acute and elective care for four of the five questions (q1: *p* = 0.0317, q2: *p* = 0.0191, q3: *p* = 0.1568, q4: *p* = 0.0031, q5: *p* = 0.038), indicating possibilities to identify differences between groups, despite the ceiling effect.

**TABLE 3:** Response frequencies (%) and number of respondents in acute and elective care.

	Response frequency (%)				*n* ^a^
Were the healthcare staff friendly to you?	Very friendly	Quite friendly	Not so friendly	Not friendly at all	
Acute	76.6	18.7	3.4	1.9	321
Elective	83.8	13.5	2.7	0.0	260
Total	79.5	16.4	3.1	1.0	581
How good were the healthcare staff at explaining things to you?	Very good	Quite good	Quite bad	Very bad	
Acute	55.1	33.4	7.0	4.5	314
Elective	64.8	27.0	4.7	3.5	256
Total	59.5	30.5	6.0	4.0	570
How good were the healthcare staff at listening to you?	Very good	Quite good	Quite bad	Very bad	
Acute	70.2	20.1	4.4	5.3	319
Elective	75.5	18.7	4.7	1.2	257
Total	72.6	19.4	4.5	3.5	576
How easy was it to ask the healthcare staff if you were wondering about something?	Very easy	Quite easy	Quite difficult	Very difficult	
Acute	59.2	24.1	11.9	4.8	294
Elective	71.4	20.6	6.9	1.2	248
Total	64.8	22.5	9.6	3.1	542
How good were the healthcare staff at helping you if you were worried or scared?	Very good	Quite good	Quite bad	Very bad	
Acute	64.7	20.7	8.7	5.9	275
Elective	73.4	19.8	4.5	2.3	222
Total	68.6	20.3	6.8	4.2	497

^a^ Total number of respondents in acute care (*n* = 324) and elective care (*n* = 262).

The Rasch analysis was based on 311 children with complete answers on all five questions. The overall model fit, assessed using the *χ* ^2^‐test of item‐trait interaction, showed no significant deviation from Rasch model expectations (*p* = 0.14). Item fit residuals (mean 0.15 std. 1.35) and person fit residuals (mean −0.24 std. 0.85) supported adequate fit. All items had a fit residuals within the ±2.5 range, and *χ* ^2^‐test for item fit were non‐significant after Bonferroni adjustment. Four children (1.3%) showed person misfit (fit residuals outside ±2.5). PCA of residuals showed that the first contrast had an eigenvalue of 1.66, below the commonly used threshold of 2.0, supporting scale unidimensionality. Local dependency was identified between items Q1 and Q3 using the more conservative limit of 0.15. No item had disordered thresholds.

Visual inspection of ICC curves indicated good correspondence between observed and expected responses across the latent trait continuum. PSI was slightly above 0.7 (0.79 including extreme persons and 0.70 excluding extreme persons). No differential item functioning (DIF) was detected for age, gender, hospital, country of birth, acute versus elective care, inpatient versus outpatient care, or short‐ versus long‐term illness.

Targeting indicated a substantial ceiling effect. Otherwise, item difficulty was reasonably aligned with the distribution of person locations, covering the central −4 to +4 logit range.

## 4 Discussion

We developed the PREM‐Ped questionnaire to capture the voices of children and adolescents. This study addresses an important gap in validated, brief, and child‐friendly patient‐reported experience questionnaires.

Many studies have already identified important aspects of PREM from the children's perspectives [16, 26]. Based on that research, we formed five key topics and used the focus expert group of children to discuss the relevance of these aspects and to ensure no important aspect was lacking. The iterative use of cognitive interviews allowed us to refine questions based on children's comprehension and response behaviour after each round. This approach ensured that the voices of the children were continuously used to improve the questions from their perspective and not only to test a final version of the questionnaire.

PREM questionnaires are often extensive, but our aim was to keep PREM‐Ped short and accessible to maximize children's ability to understand the questions and respond independently. Consequently, the questionnaire was limited to the domains that children identified as most important. Although additional aspects, such as the physical environment, were suggested, the included domains were considered most essential for children to feel satisfied and safe during their hospital visit.

We initially included a more direct question on patient participation, but it proved difficult for children to understand. The cognitive interviews suggested that several of the final items represent important prerequisites for also participation and shared decision‐making. In particular, the item on whether HCP listened to the child appeared to capture key aspects of participation. The items do not imply that children can opt out of necessary procedures they don't want or like to do; rather, it reflects the importance of HCPs listening to children, adapting their approach when possible, and providing support when children feel scared or worried.

When designing patient‐reported experience measures for children, several methodological factors must be carefully considered. Age‐related cognitive differences warrant attention. The fact that some children chose to answer *Don't know* when the questions were read out loud with the five‐point scale may be explained by a recency effect. This tends to become more pronounced when the number of response options increases, as respondents are less able to retain all alternatives in working memory [27]. To minimize that problem and make the questions feasible also to be read out to younger children, the response options were restricted to four.

Another challenge is to ensure that responses reflect care quality rather than discomfort associated with necessary procedures. The questions are therefore developed to specifically address; for example, the HCP's ability to help the child if feeling scared. Our interviews indicate that children generally differentiate between HCPs' behaviour and procedural discomfort, but this needs to be confirmed in more interviews in different settings.

The use of value‐laden terms, such as “good”, is generally avoided in survey questions; however, when directed to children, the absence of such terms makes the questions too abstract [28]. The iterative rounds of the cognitive interviews contributed to phrasings that would feel natural to ask a child not only in a questionnaire, but also in a real‐life conversation.

The question about asking questions may be difficult for children to know how to respond if they had never asked a question. The question is phrased in a way that it is possible to answer hypothetically from a general feeling that it would have been easy *if* they would have wanted to ask a question; for example, by HCPs inviting questions or building trust by communication with the child. There is, however, a risk that children who feel very shy and feel they would never dare to ask questions may respond “Very difficult”. It is possible that this issue is more pronounced in acute care, where there may be more brief encounters during which the child does not know the HCPs.

Previous research highlights that most paediatric PREMs rely on proxy responses from caregivers, which may not accurately reflect the child's perspective [16, 26]. The PREM‐Ped design was developed to facilitate for children to independently respond to the questions in line with UNICEF [7] principles emphasizing children's right to express their views in matters affecting them. In such trade‐offs, prioritizing the child's perspective and accessibility has been considered more important than maximizing psychometric properties.

The Rasch analysis showed good psychometric properties, except the ceiling effect. When using a conservative limit there was some indication of local dependency between the question about *kindness* and *listen* which may indicate a more empathic dimension. Since the indicators for unidimensionality and adequate item fit, all five items still seem to contribute well to the overall scale. Ceiling effects are common in patients' satisfaction measurement tools and are difficult to eliminate by expanding the response options to a higher level of satisfaction since many patients will always choose the best option to express thankfulness for being helped by the healthcare [3]. The pilot study was conducted at two sites at Astrid Lindgren Children's Hospital within Karolinska University Hospital, a large specialized tertiary paediatric hospital with international recognition [29]. This may have contributed to a higher ceiling effect compared to settings where care is not specifically designed for children.

Known‐group validity was assessed by comparing perceived experiences in acute versus elective care, based on the a priori hypothesis that acute care would be associated with lower satisfaction. The results demonstrated statistically significant differences between the two care settings, with four out of five items showing significant differences. These findings indicate that the PREM‐Ped is capable of discriminating between different care settings that are theoretically expected to differ, thereby strengthening evidence for its construct validity.

Benchmarking plays a crucial role in healthcare, as it allows providers to compare their quality of care against established standards and identify areas for improvement [11, 30]. We developed the PREM‐Ped to support benchmarking of children's healthcare experiences across care settings. The PREM‐Ped can be used to identify domains where children report suboptimal experiences, to guide local improvement efforts (e.g., communication, involvement in care, etc.). It also enables comparisons between different care settings and units, thereby facilitating shared learning and dissemination of best practice. This is particularly relevant given substantial international and intra‐national variation in healthcare quality improvement and in how patient experiences are measured. Further testing and validation in other cultural and linguistic settings are needed to support broader comparability, work that is currently ongoing in collaboration with several countries in the European Children's Hospital Organization (ECHO).

## 5 Strengths and Limitations

A key strength of this study is the participatory development process, including iterative testing with children. A consequence of this is, however, that some terms or phrases that were not changed are tested in many interviews whereas the final changes are tested in the last round. The use of Sweden's national digital platform, 1177, enables questionnaires to be delivered to children aged 13–17 years at a timepoint close after a visit (24 h). However, the PREM‐Ped administered via this platform did not allow respondents to skip items, which may have forced some participants to select an unfounded answer or leave the survey. Additional limitations include reliance on a single focus group and the pilot's restriction to one healthcare region, which may limit the diversity of perspectives and affect generalizability.

Future research should examine psychometric properties in larger samples and assess the utility of AI‐assisted analysis for open‐ended responses. Future research should also include development of PREM‐Caregivers.

## 6 Conclusion

This study presents the PREM‐Ped questionnaire, a brief and child‐friendly instrument designed to capture children's own healthcare experiences, addressing a gap in paediatric patient‐reported experience measurement. Initial psychometric evaluation demonstrated satisfactory measurement properties and supported known‐group validity, with the instrument discriminating between acute and elective care contexts.

PREM‐Ped enables benchmarking of children's healthcare experiences across settings, thereby supporting systematic, child‐centred quality improvement. Overall, PREM‐Ped represents a robust step toward inclusive, patient‐centred evaluation of paediatric healthcare.

## Author Contributions

**C. Bartholdson:** conceptualization, funding acquisition, writing – original draft, methodology, writing – review and editing, formal analysis, project administration, investigation, validation. **J. Granhagen Jungner:** writing – original draft, methodology, writing – review and editing, formal analysis, funding acquisition, conceptualization, investigation, validation, visualization. **E. Broström:** conceptualization, funding acquisition, writing – original draft, methodology, writing – review and editing, formal analysis, project administration, resources, data curation, investigation, validation, visualization. **S. Norgren:** writing – review and editing, resources, project administration, data curation. **M. Wenemark:** methodology, formal analysis, writing – review and editing, writing – original draft, investigation, validation, visualization, software.

## Funding

The study was funded by research grants from Sällskapet Barnavård, Stockholm, Sweden, and Karolinska Institutet Research Foundation Grants, 2018‐02217, Stockholm, Sweden.

## Ethics Statement

The study was conducted in accordance with the principles outlined in the Declaration of Helsinki. Ethical approval was obtained from the Ethical Review Authority in Sweden (Dnr 2023‐07271‐02). All participants in the focus group and cognitive interviews received both written and oral information about the study. Written informed consent was obtained from parents or legal guardians for participants under 15 years, while participants aged 15 years and older provided consent themselves. In the pilot study, answering the questionnaire was regarded as providing consent which was clearly stated in the invitation to the survey.

## Conflicts of Interest

The authors declare no conflicts of interest.

## Data Availability

The data that support the findings of this study are available on request from the corresponding author. The data are not publicly available due to privacy or ethical restrictions.
